# Age- and sex-associated effects of C18 ceramide sphingolipids on osteoclastogenesis in experimental models of Gulf War Illness

**DOI:** 10.1016/j.biopha.2025.118418

**Published:** 2025-08-18

**Authors:** Chiaki Yamada, Amilia Nusbaum, Natasha Sanz, Hawra AlQallaf, Benjamin A. Cameron, Lubov Nathanson, Clark T. Barco, Nancy Klimas, Alexandru Movila

**Affiliations:** a Department of Biomedical Sciences and Comprehensive Care, Indiana University School of Dentistry, Indianapolis, IN, USA; b Indiana Center for Musculoskeletal Health, Indiana University School of Medicine, Indianapolis, IN, USA; c Richard L. Roudebush VA Medical Center, Indianapolis, IN, USA; d Department of Periodontology, Indiana University School of Dentistry, Indianapolis, Indiana, USA; e Institute for Neuro Immune Medicine, Dr. Kiran Patel College of Osteopathic Medicine, Nova Southeastern University, Fort Lauderdale, FL, USA; f Geriatric Research, Education, and Clinical Center, Veterans Affairs Medical Center, Miami, FL, USA

**Keywords:** Osteoclastogenesis, Gulf War illness, Toxins, Ceramide, In vitro

## Abstract

Approximately 60 % of Gulf War Illness (GWI) cases are correlated with toxic exposure to permethrin (PER) and pyridostigmine bromide (PB) in Veterans. Among the known hallmarks of GWI, pathological changes in bone of Veterans with GWI are poorly understood due to the lack of relevant experimental models of osteoclastogenesis. Emerging metabolomic studies have reported that GWI symptoms are positively correlated with the accelerated prevalence of ceramide sphingolipids in the serum. According to a secondary analysis of publicly available targeted metabolomic datasets, the area under the curve (AUC) value of C18:0 ceramide was significantly elevated by more than 56 % in the serum of male GWI Veterans compared to non-veteran healthy controls. Using mouse bone marrow-derived osteoclast precursors from young (2-month-old) and aged (22-month-old) mice, our observational studies confirmed that C18:0 and C18:1 significantly accelerated RANKL-primed osteoclastogenesis *in vitro*. Furthermore, C18:0 ceramide increased RANKL-primed osteoclast formation in aged, but not young male osteoclast precursors exposed to PB or PER *in vitro*. In contrast, a mixture of C18:0 and C18:1 with PB reduced the number of osteoclasts from young female mice *in vitro*. In addition, C18:1 diminished RANKL-primed osteoclastogenesis in young male as well as young and aged female mouse osteoclast precursors in the presence of PER *in vitro*. As Veterans with GWI rapidly approach the senior 65+ age range, further studies are warranted to evaluate the potential link between Gulf War toxic exposure and ceramide sphingolipids in age- and sex-associated osteoclastogenesis.

## 1. Introduction

Gulf War Illness (GWI) is a chronic toxic chemical-exposed disorder affecting over 25 % of Veterans who served in the Persian Gulf War between 1990 and 1991 [1,2]. Although the identification of a single etiological GWI toxic agent remained elusive, most of the studies agree that 60 % of soldiers were exposed to insecticide spray containing Permethrin (PER) and anti-nerve agent pills with Pyridostigmine Bromide (PB) at significantly higher doses than recommended, which correlates in part with GWI symptoms [3,4].

According to the Kansas criteria and the Center for Disease Control (CDC) health condition criteria for Gulf War Veterans, the spectrum of GWI symptoms involves sleep and fatigue disturbances, cognitive and mood changes, gastrointestinal and musculoskeletal changes [2,5,6]. Alarming epidemiological data indicate that the surviving cohort of Gulf War Veterans is rapidly reaching the senior (65+) age [7]. In recent years, we have significantly advanced our understanding of the GWI hallmark features of the age-associated changes in the central neurological and gastrointestinal systems [3,8]. However, there is still limited knowledge of the effect of advanced age on bone changes in response to GWI toxins. Using trans-iliac bone samples, a cross-sectional clinical study reported that Veterans of the Gulf War have reduced bone formation and accelerated bone osteolysis [9]. Furthermore, diminished bone mineral density in the senior cohort of Veterans with GWI symptoms was recently observed [10]. Using knowledge of age-associated bone loss and health issue hallmarks observed in Veterans with GWI, a hypothesis-driven paper further proposed a potential crosstalk between osteoporosis and GWI-toxic exposure in Veterans at a senior age [11], emphasizing the immediate need to increase our understanding of the mechanisms of bone osteolysis in Veterans exposed to GWI toxins.

It is widely accepted that bone osteolysis is linked with accelerated formation and activity of osteoclasts, unique bone resorbing cells [12, 13]. Fusion of osteoclast precursors and formation of functional multinucleated osteoclasts from monocyte/macrophage lineage is critically controlled by the Receptor Activator of Nuclear factor-κB Ligand (RANKL). Importantly, we recently detected elevated prevalence of monocyte population in peripheral blood mononuclear cells obtained from Veterans with GWI compared to healthy controls [14]. Additionally, several pro-inflammatory factors associated with inflammatory osteoclastogenesis, e.g., High Mobility Group Box 1, Interleukin-6, and C-reactive protein, were detected in the serum of Veterans with GWI [15-18]. Using a single-cell RNA-sequencing assay, we also observed that mononuclear cells from Veterans with GWI have elevated expression of genes engaged in cellular response to lipids[14].

Accumulated lines of evidence further indicate that bioactive sphingolipids, particularly ceramide species, play an essential role in the promotion of age-associated bone osteolysis [19]. Using monocytes as osteoclast precursors, we also reported that ceramide species accelerate RANKL-primed osteoclastogenesis *in vitro* [20]. Ceramide sphingolipids serve as a downstream signaling cascade in the cell’s inflammatory responses mediated by High Mobility Group Box 1, Interleukin-6, and C-reactive protein [21-23].

While it has also been demonstrated that the levels of ceramide sphingolipids increase in the plasma of GWI veterans[24,25], another published cross-sectional clinical study reported reduced osteoclast numbers in bone biopsies of GWI veterans compared to healthy controls [26]. In addition, there is limited knowledge about the impact of ceramides on osteoclastogenesis in the context of GWI toxin exposure due to the lack of an experimental *in vitro* model of RANKL-primed osteoclastogenesis. Therefore, the initial goal of the study was to select promising ceramide candidates and GWI toxins for developing an experimental model of osteoclastogenesis, which will be relevant to study bone health in Veterans with GWI. Using these models, we further aimed to identify whether GWI-relevant ceramide species inhibited RANKL-primed osteoclastogenesis in response to GWI toxins, PB and PER. This study also emphasized the impact of biological sex and age on osteoclastogenesis in the context of exposure to GWI toxins.

## 2. Materials and methods

### 2.1. Re-analysis of targeted metabolites database created using the serum of male Veterans with GWI and healthy controls

Given that a cross-sectional study reported an elevated prevalence of some ceramide species in the serum of GWI veterans [25], we have re-analyzed a publicly available database of targeted metabolites with a particular focus on the specific ceramide species with known effect on RANKL-primed osteoclastogenesis.

The database represents the area under the curve (AUC) of 358 targeted broad-spectrum metabolites, which are virtually associated with 44 biochemical pathways identified by LC-MS/MS mass spectrometry. The sample detailed screening protocol and database organization were described in detail elsewhere [25]. It was derived from the serum of 40 male participants, including 20 veterans with GWI symptoms (average age: 49 ± 1.8 years) and 20 non-veteran controls (average age: 48 ± 1.8 years) without GWI symptoms. Veterans with GWI met both the Kansas and the Centers for Disease Control (CDC) diagnostic criteria for GWI. In contrast, the control group consisted of non-veteran volunteers who met neither the Kansas nor the CDC diagnostic criteria for GWI, nor any Kansas exclusion criteria for GWI, e.g., lupus or multiple sclerosis.

### 2.2. Preparation of Sphingolipid and GWI toxin solutions

Stock solutions of C18:0 (d18:1/18:0) and C18:1 (d18:1/18:1(9Z) (both from Cayman) sphingolipid ceramides were prepared in DMSO and stored at −20°C. The stock solutions of GWI toxins, PER (Enzo) and PB (Acros Organics), were prepared using 100 % ethanol, according to the manufacturer’s recommendations. The ceramides and GWI toxins were further dissolved in α-MEM media (Corning) supplemented with 10 % FBS (Biotechne) for biological experiments. The final concentration of DMSO and ethanol was less than 0.01 %. Control groups of cells were exposed to DMSO and ethanol-containing solutions.

### 2.3. Mouse Bone Marrow mononuclear cells isolation and osteoclast proliferation assay

Young (2-month-old) and aged (22-month-old) male and female C57BL/6 mice were obtained from the NIA rodent colony. After mouse euthanasia, we collected femur and tibia for the isolation of bone marrow mononuclear cells using gradient centrifugation in Histopaque^®^-1083 (Sigma Aldrich). Then, the cells were seeded into a 96-well plate at a density of 1 × 10^5^ cells/well in macrophage proliferation media for 3 days. The macrophage proliferation media contained 20 ng/ml of recombinant mouse M-CSF (BioLegend) in α-MEM medium (Corning) supplemented with 10 % FBS (Biotechne), 1 % penicillin/ streptomycin, 1 % L-glutamine, and 1 % MEM-NEAA (all from Corning). After preincubation with macrophage proliferation media, mouse soluble recombinant RANKL protein (10 ng/ml; BioLegend) was added to the culture media. In addition, some groups of cells were exposed to a mixture of GWI toxins, PB or PER (1 μM of each), with ceramide C18:0 or C18:1 species (01, 1, or 10 μM). Osteoclasts in the culture were identified using the leukocyte acid phosphatase (tartrate-resistant acid phosphatase [TRAP]) kit (Sigma-Aldrich) at day 5 after RANKL stimulation. TRAP-positive multinuclear cells, containing 3 nuclei or more, were counted microscopically, and the results were expressed as the number of cells per well.

### 2.4. Statistical analysis and data presentation

**Fig. 1.**
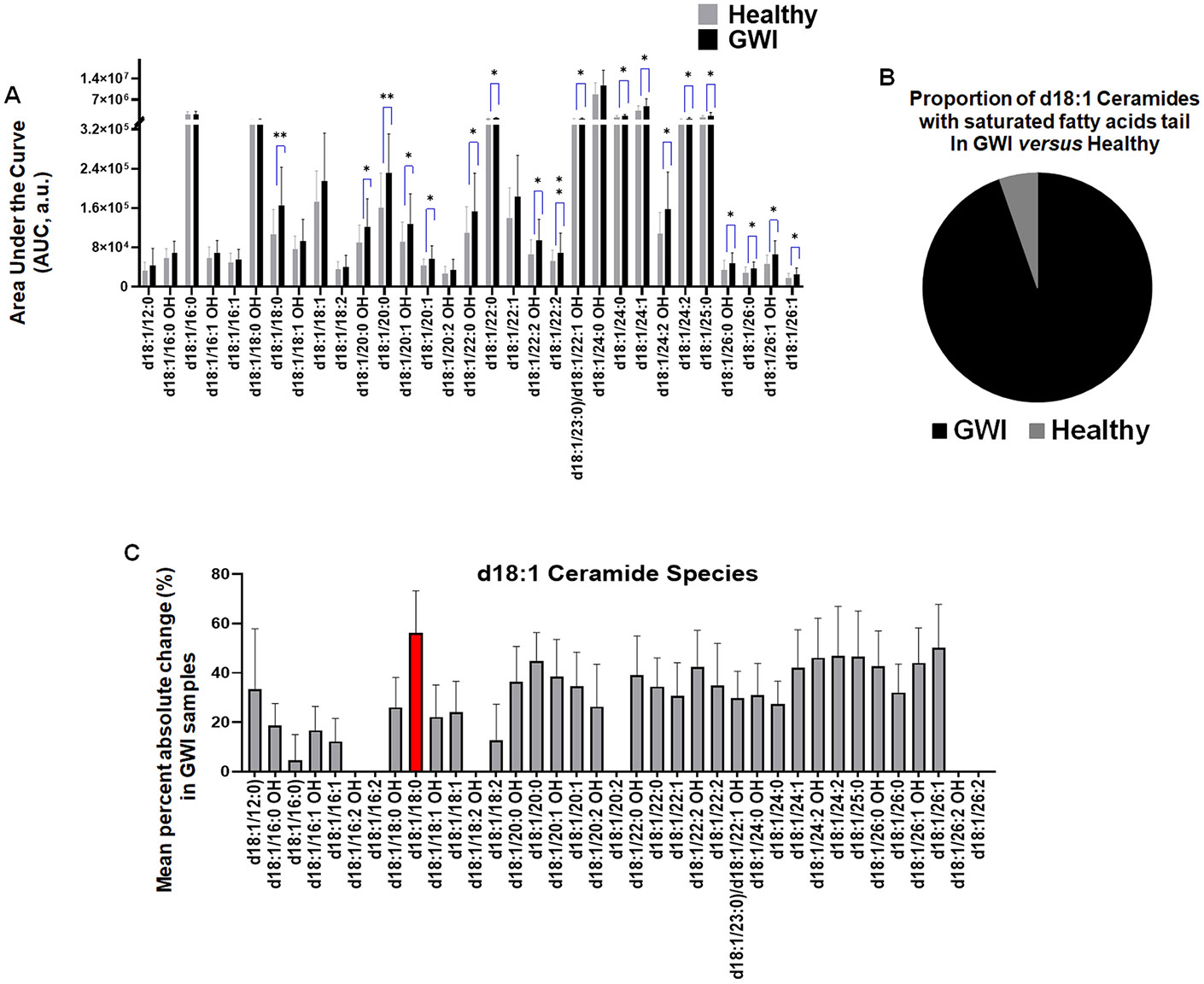
Prevalence of d18:1 ceramide species in the serum of veterans with Gulf War Illness (GWI) symptoms and healthy controls. A: AUC of d18:1 ceramide species with saturated and unsaturated fatty acid tails detected in the serum of veterans with GWI and healthy controls using a targeted metabolomic assay. B: The pie chart compares the total levels of ceramide species with saturated fatty acid tails between Veterans with GWI symptoms and healthy controls, based on the result shown in panel A. According to the metabolomic results (see panel A), the total amount of ceramide with saturated fatty acids significantly increased in the serum of Veterans with GWI. C: The graph displays an absolute percent change (±SEM) of different ceramide species in the serum of Veterans with GWI relative to the adjusted values of healthy controls. The d18:1/18:0 (=C18:0) ceramide is the most accelerated bioactive sphingolipid detected in the serum of Veterans with GWI using a targeted metabolomic assay. Results were obtained based on the 37 ceramide species available in the open source targeted broad-spectrum metabolomic database [25]. The student’s *t*-test was used to identify statistical significance. n = 40 samples, including 20 from Veterans with GWI and 20 healthy controls. *p < 0.05.

**Fig. 2.**
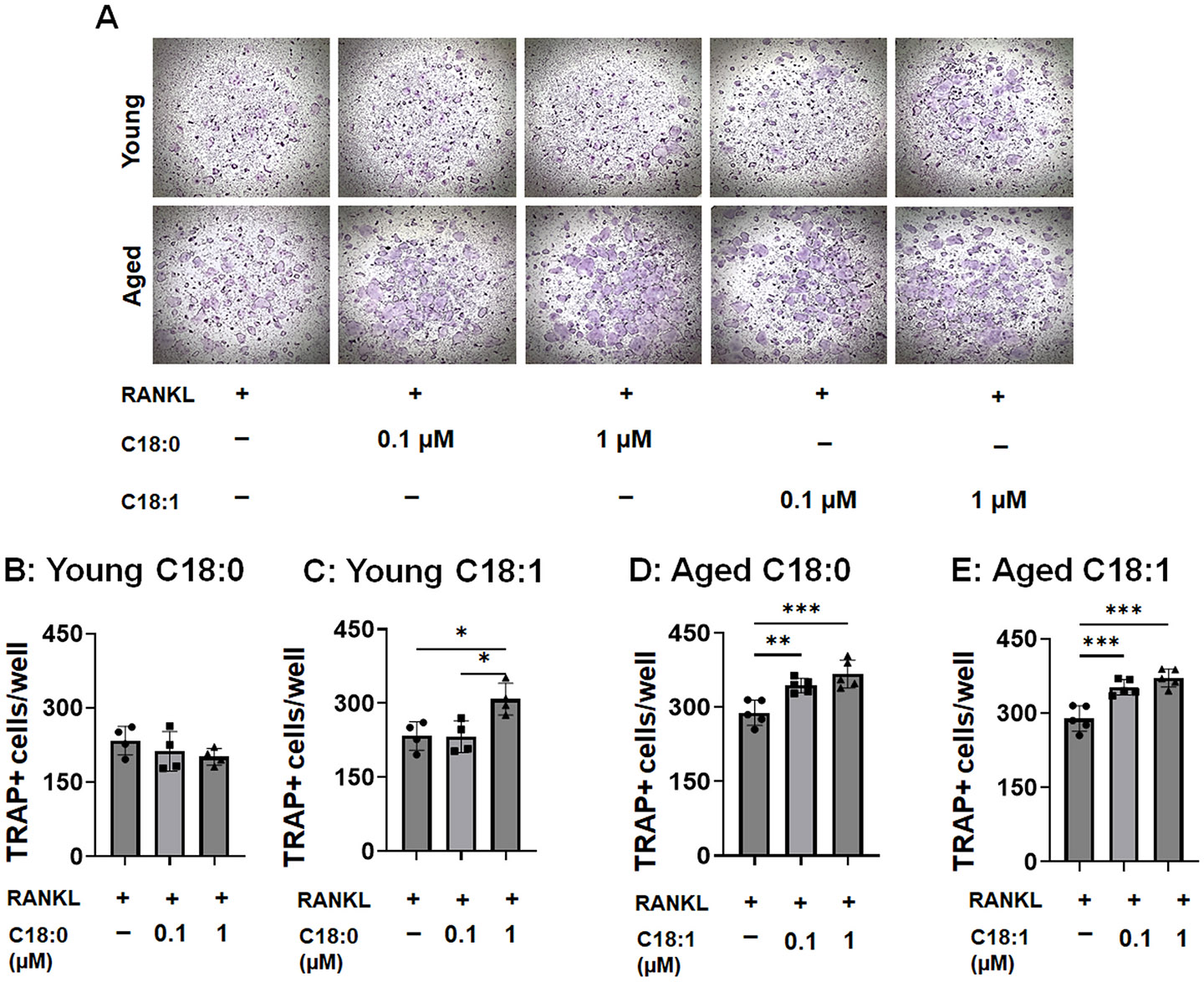
Ceramide C18:0 and C18:1 species promote RANKL-primed osteoclastogenesis in the context of aging *in vitro*. A: Representative images of TRAP+ osteoclast-like cells exposed to RANKL alone or in the presence of C18:0 or C18:1 ceramide sphingolipids. B & C: Quantifying the number of TRAP+ osteoclast-like multinucleated cells generated from young and aged osteoclast precursors in response to RANKL alone or combined with C18:0 ceramide. D & E: Quantification of the number of TRAP+ osteoclast-like multinucleated cells generated from young and aged osteoclast precursors in response to RANKL alone or in combination with C18:1 ceramide. To induce osteoclastogenesis, bone marrow-derived mononuclear cells were isolated from young (2-month-old) and aged (22-month-old) male C57/B6 mice. Then, we first proliferated them to macrophages using the M-CSF recombinant mouse protein. After 3 days, cells were primed with a mixture of M-CSF/RANKL alone (=shown as RANKL) or in the presence or absence of different concentrations of C18:0 or C18:1 ceramide species. n = 4–5 samples/condition. *p < 0.05, **p < 0.01, and ***p < 0.001 based on the ANOVA with Tukey’s post-hoc test.

**Fig. 3.**
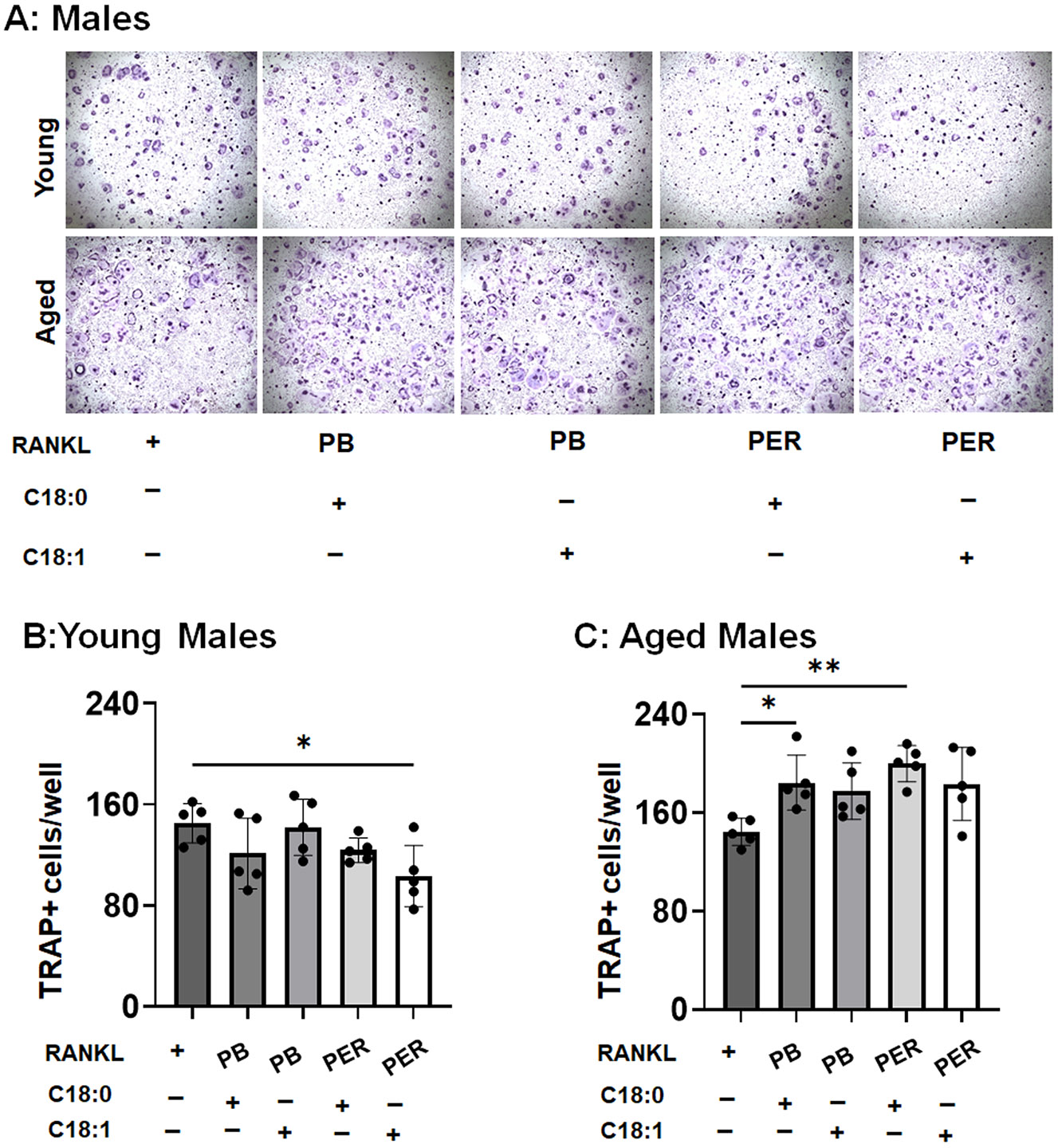
Age-associated effects of C18:0 and C18:1 ceramide species on male RANKL-primed osteoclastogenesis in response to GWI toxins, pyridostigmine bromide (PB) and permethrin (PER) *in vitro*. A: Representative images of TRAP+ osteoclast-like cells exposed to C18:0 or C18:1 ceramide species in the presence of PB or PER. Quantification of the number of TRAP+ osteoclast-like multinucleated cells generated from young (B) and aged (C) osteoclast precursors in response to RANKL alone or combination with C18:0 or C18:1 ceramide (1 μM of each) and GWI toxins, PB or PER (1 μM of each). To induce osteoclastogenesis, bone marrow-derived mononuclear cells were isolated from young (2-month-old) and aged (22-month-old) male C57/B6 mice. Then, we differentiated them into macrophages using the recombinant M-CSF protein. After 3 days, cells were stimulated with a mixture of M-CSF/RANKL alone (=shown as RANKL) or in the presence or absence of C18:0 or C18:1 ceramide species and PB or PER. TRAP staining was done after 5 days of stimulation with RANKL. n = 4–5 samples/condition. *p < 0.05 and **p < 0.01 based on the ANOVA with Tukey’s post-hoc test.

**Fig. 4.**
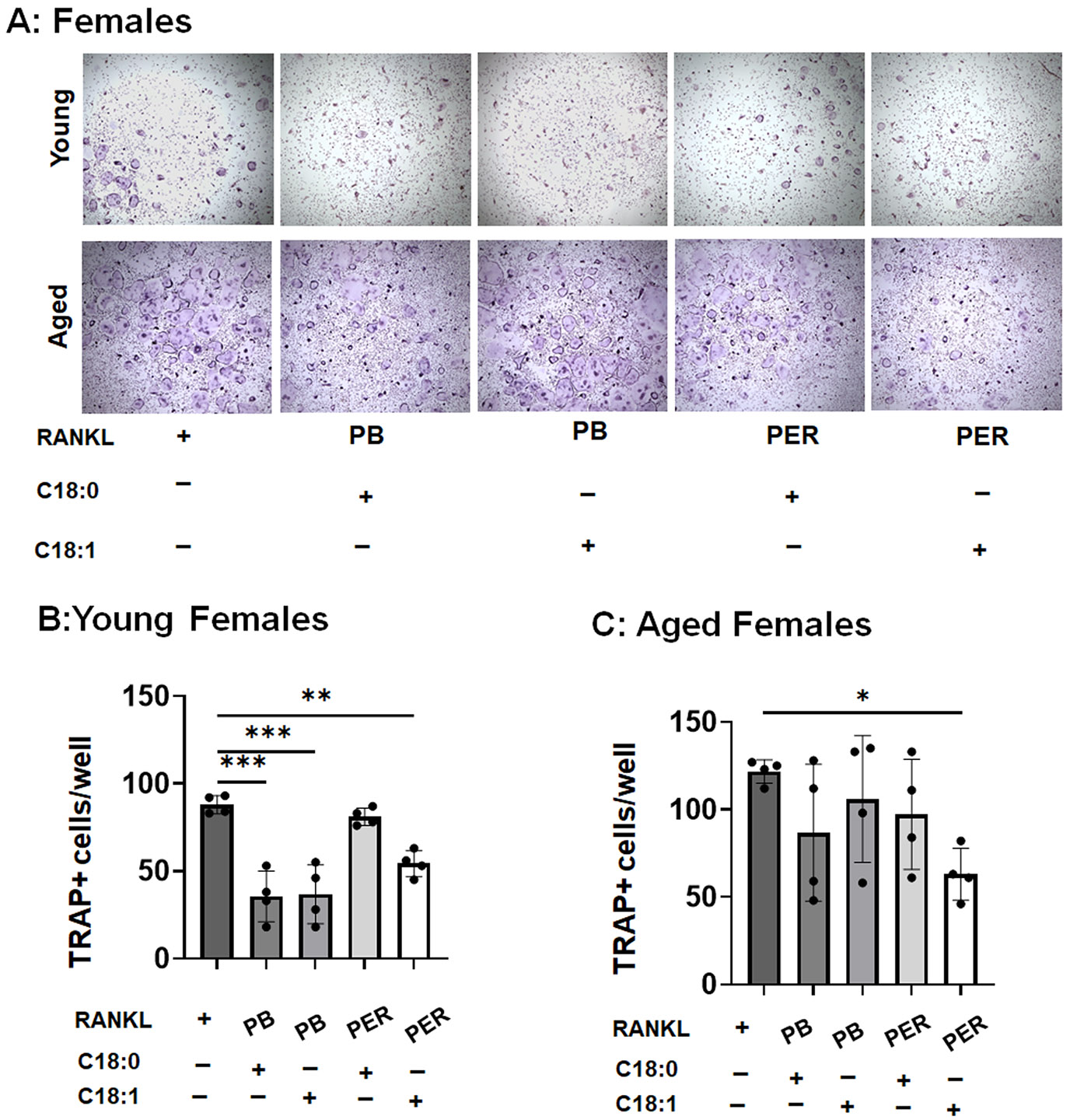
Age-associated effects of C18:0 and C18:1 ceramide species on female RANKL-primed osteoclastogenesis in response to GWI toxins, pyridostigmine bromide (PB), and permethrin (PER) *in vitro*. A: Representative images of TRAP+ osteoclast-like cells exposed to C18:0 or C18:1 ceramide species (1 μM of each) in the presence of PB or PER (1 μM of each). Quantification of the number of TRAP+ osteoclast-like multinucleated cells generated from female young (B) and aged (C) osteoclast precursors in response to RANKL alone or in combination with C18:0 or C18:1 ceramide and GWI toxins, PB or PER. To induce osteoclastogenesis, bone marrow-derived mononuclear cells were isolated from young (2-month-old) and aged (22-month-old) male C57/B6 mice. Then, we proliferated them to macrophages using the M-CSF recombinant protein. After 3 days, cells were stimulated with a mixture of M-CSF/RANKL alone (=shown as RANKL) or in the presence or absence of C18:0 or C18:1 ceramide species and PB or PER. TRAP staining was done after 5 days of stimulation with RANKL. n = 4–5 samples/condition. *p < 0.05, **p < 0.01, and ***p < 0.001 based on the ANOVA with Tukey’s post-hoc test.

The ceramide species-specific AUC values were used to compare statistical significance between veterans with GWI and healthy controls using a Student’s *t*-test (Fig. 1A). Clinical-based metabolomic data are represented as the mean percent absolute change ± standard error of the mean (SEM). Error analysis and propagation for metabolomic data analysis were based on the recommendations published elsewhere [27]. For *in vitro* studies, ANOVA with post hoc Tukey’s test was used to compare the impact of ceramide and GWI toxins, PB and PER, on osteoclastogenesis *in vitro* (Figs. 2-4). In vitro data are represented as the mean number of TRAP+ osteoclasts ± standard deviation. In addition, the percent absolute change between the control group treated with RANKL alone and the experimental group exposed to RANKL in combination with ceramide species and GWI toxins was calculated. A *p* < 0.05 was considered statistically significant. The data were analyzed using the PAST 4.03 and PRISM statistical software.

## 3. Results and discussion

### 3.1. Elevated levels of C18:0 ceramide in the serum of veterans with GWI

To better understand whether ceramide sphingolipids are elevated in GWI, we have initially compared the prevalence of ceramides with saturated and unsaturated fatty acid tails in the serum of GWI using an open-source database. The basic structure of ceramide sphingolipids consists of sphingosine (d18:1), fatty acid tails, and a polar head group. We identified a database containing ceramide sphingolipid specimens, consisting of sphingosine (d18:1) with 37 different fatty acid tails, detected in the serum of veterans with GWI symptoms and healthy controls in males by targeted metabolomic mass-spectrometry assay [25]. To the best of our knowledge, the d18:1 ceramide specimens with the following fatty acid tails were significantly increased in veterans with GWI symptoms: C18:0, C20:0, C20:1-OH, C20:1, C22:0, C22:2, C23:0, C24:0, C24:1, C24:2, C25:0, C26:0-OH, C26:0, C26:1-OH, and C26:1 (Fig. 1A). Of note, the GWI veterans have elevated levels of the ceramides with the saturated fatty acid tail compared to healthy controls (Fig. 1B). Based on the secondary database analysis, we further identified that the C18:0 ceramide (d18:1/18:0) was elevated by 156.1 % in the serum of GWI veterans compared to healthy controls making it as the most abundant ceramide species (Fig. 1C). Our data confirms a recently published study detected elevated prevalence of C18:0 ceramide in ocular meibum of Veterans with GWI [28]. Importantly, C18:0 ceramide (d18:1/18:0) with a saturated fatty acid tail and its C18:1 unsaturated variant (d18:1/18:1) are both linked to age-associated bone osteolysis [29]. Furthermore, it was also recently demonstrated that GWI toxins experience accelerated cellular aging [30]. Therefore, it is plausible that C18:0 ceramide is one of the potential bioactive sphingolipids engaged in accelerated aging age-associated health hallmarks in Veterans with GWI. It is important to emphasize that the GWI metabolomic database was created based on the targeted broad-spectrum metabolomic assay, representing one of the study’s limitations. Another limitation is that the sample size was only obtained from 20 GWI and 20 healthy males. Therefore, analyzing the serum of male and female Veterans with GWI using an untargeted metabolomic assay will significantly improve our knowledge about the impact of ceramide sphingolipids in the GWI pathology.

Since C18:0 ceramide production is controlled by the ceramide synthetase 1 (CERS1) in the smooth endoplasmic reticulum [12], we may speculate that veterans with GWI may have accelerated activity of CERS1. Furthermore, CerS1-derived C18:0 ceramide species in skeletal muscle promoted obesity-induced insulin resistance [31]. It was further recently suggested that inhibition of CERS1 may serve as an attractive therapeutic regimen for treating metabolic diseases, which are also linked to severe symptoms of GWI [32,33].

### 3.2. C18:0 ceramide accelerated physiological RANKL-primed osteoclastogenesis in vitro

Several studies reported that ceramide plays an essential role in bone metabolism [34]. Furthermore, C18:0 and C18:1 ceramide species are correlated with bone resorption markers in the context of aging [29]. First, we tested the impact of C18:0, C18:1, and C16:0 ceramide species on physiological osteoclastogenesis in the absence of GWI toxins *in vitro* (Fig. 2A). Our data indicate that only C18:1 ceramide, but not C18:0, significantly accelerated the number of TRAP+ osteoclast-like cells from RANKL-primed young male osteoclast precursors by 33.0 % (Fig. 2B, C). In contrast, both C18:0 and C18:1 ceramide promoted the accumulation of TRAP+ osteoclast-like cells from male aged osteoclast precursors in response to RANKL by 27.1 % and 28.4 %, respectively (Fig. 2D, E). No significant effects of C16:0 ceramide on the number of TRAP+ osteoclast-like cells were observed in young and aged RANKL-primed male osteoclast precursors *in vitro* (data not shown). Therefore, these data agree that both C18:0 and C18:1 ceramide species accelerate age-associated physiological osteoclastogenesis in RANKL-primed osteoclast precursors.

### 3.3. The opposite sex- and age-associated effect of ceramide species on osteoclastogenesis in response to GWI toxins

Among the known GWI toxins, PER and PB are prominent chemical toxins to study experimental hallmarks of GWI [35]. Since limited knowledge exists about the age-associated osteoclastogenesis in response to GWI, we next employed the GWI toxins, e.g. PB and PER, to understand the impact of C18:0 and C18:1 ceramide species on RANKL-primed osteoclastogenesis in the context of sex and age.

Using osteoclast precursors isolated from young male mice, we surprisingly detected that a mixture of C18:1, but not C18:0, with PER significantly inhibited the formation of TRAP+ osteoclast-like cells in response to RANKL by 28.8 % *in vitro* (Fig. 3A and B). Furthermore, no or little effect of a mixture of C18:1 with PER on RANKL-primed osteoclastogenesis was detected in aged osteoclast precursors in vitro (Fig. 3A and C). In contrast, C18:0 significantly promoted the formation of aged male TRAP+ osteoclast-like cells in the presence of PB or PER by 26.7 % and 38.3 %, respectively (Fig. 3A and C).

While the Gulf War women Veterans represent an increasing proportion of GWI cases compared to their male Veteran counterparts in U. S. military history [36], we have limited knowledge about the emerging effects of ceramide species on age-associated osteoclastogenesis as well as bone pathology in female Gulf War Veterans. According to the sex-specific screening of plasma lipid profiles, the total ceramide species were accelerated in female Veterans with GWI, but not in males, compared to healthy individuals [24]. Therefore, we finally compared whether C18:0 or C18:1 ceramides demonstrate any age-associated effects on female RANKL-primed osteoclast precursors *in vitro*.

We observed that the number of TRAP+ osteoclast-like cells was accelerated in young and aged female osteoclast precursors exposed to RANKL (Fig. 4A-C). In contrast, our study demonstrated that combining C18:0 or C18:1 ceramide with PB has reduced the number of TRAP+ osteoclast-like cells obtained from young RANKL-primed osteoclast precursors by 59.7 % or 58.2 %, respectively. However, a mixture of C18:1 with PER ceramide inhibited the formation of TRAP+ osteoclast-like cells obtained from young and aged RANKL-primed osteoclast precursors by 38.3 % and 48.2 %, respectively (Fig. 4A-C). Altogether, these results indicated that C18:0 and C18:1 inhibit RANKL-primed osteoclastogenesis in young and aged female osteoclast precursors in the presence of GWI toxins, PB and PER, *in vitro*.

Several *in vitro* studies have reported on the differentiation process, revealing variations in osteoclast formation between sexes, as well as its influence by age and culture conditions in response to RANKL [37-39]. Furthermore, it was also demonstrated that female bone marrow cells differentiated faster into osteoclasts than male bone marrow cells using non-GWI models[38]. According to our results, a mixture of GWI toxins with ceramide species inhibited RANKL-primed osteoclastogenesis. Since diminished osteoclastogenesis can negatively impact bone fracture healing and repair by hindering the resorption of old or damaged bone tissue[40], it is plausible that Veterans with GWI may have issues with bone health due to inhibited osteoclastogenesis. We must emphasize that we used mouse osteoclast precursors exposed to GWI toxins and ceramide species. Thus, confirming data from these studies using osteoclast precursors from Veterans with GWI and non-deployed healthy controls is essential. Furthermore, this study reveals distinct sex- and age-associated effects of C18 ceramide species and GWI toxins, PB and PER, on the promotion of RANKL-primed osteoclastogenesis *in vitro*. Although males predominantly represent the Veteran population from the 1990–1991 Gulf War conflict, our findings underscore the immediate need to study bone health in both male and female Veterans with GWI.

## 4. Conclusion

This study demonstrated that ceramide species containing saturated fatty acids are significantly elevated in the serum of veterans with GWI. Notably, the C18:0 ceramide emerged as the only species elevated by more than 56 % in the context of GWI pathology. We also developed a novel model of GWI-associated osteoclastogenesis using PB and PER toxins. Utilizing this model, we investigated the age- and sex-dependent effects of C18 ceramide species, e.g., C18:0 and C18:1, on promoting RANKL-primed osteoclast differentiation *in vitro*. In response to GWI toxins, we found that PER and PB differentially influenced ceramide-induced osteoclastogenesis. These distinct effects may serve as a potential diagnostic tool for more accurately identifying GWI-related pathology. Furthermore, this study confirmed previous findings that the number of osteoclasts may be reduced in the bone tissue of Veterans with GWI [41]. Therefore, further studies are warranted to elucidate the potential link between GWI-related toxic exposure and bone health, with particular emphasis on the impact of senior age and biological sex differences.

## Data Availability

Data will be made available on request.
